# ViscumTT induces apoptosis and alters IAP expression in osteosarcoma in vitro and has synergistic action when combined with different chemotherapeutic drugs

**DOI:** 10.1186/s12906-016-1545-7

**Published:** 2017-01-07

**Authors:** Susann Kleinsimon, Gwenda Kauczor, Sebastian Jaeger, Angelika Eggert, Georg Seifert, Catharina Delebinski

**Affiliations:** 1 Department of Pediatric Oncology/Hematology, Otto-Heubner-Centre for Pediatric and Adolescent Medicine (OHC), Charitém, Universitätsmedizin, Augustenburger Platz 1, 13353 Berlin, Germany; 2 Birken AG, Streiflingsweg 11, 75223 Niefern-Öschelbronn, Germany

**Keywords:** Mistletoe, *Viscum album* L, Triterpenic acids, Osteosarcoma, Apoptosis, Chemotherapeutic drugs

## Abstract

**Background:**

Osteosarcoma is the most common bone tumor and is associated with a poor prognosis. Conventional therapies, surgery and chemotherapy, are still the standard but soon reach their limits. New therapeutic approaches are therefore needed. Conventional aqueous mistletoe extracts from the European mistletoe (*Viscum album* L.) are used in complementary cancer treatment. These commercial extracts are water-based and do not include water-insoluble compounds such as triterpenic acids. However, both hydrophilic and hydrophobic triterpenic acids possess anti-cancer properties. In this study, a whole mistletoe extract viscumTT re-created by combining an aqueous extract (viscum) and a triterpene extract (TT) was tested for its anti-cancer potential in osteosarcoma.

**Methods:**

Two osteosarcoma cell lines were treated with three different mistletoe extracts viscum, TT and viscumTT to compare their apoptotic potential. For this purpose, annexin/PI staining and caspase-3, −8 and −9 activity were investigated by flow cytometry. To determine the mechanism of action, alterations in expression of inhibitors of apoptosis (IAPs) were detected by western blot. Apoptosis induction by co-treatment of viscum, TT and viscumTT with doxorubicin, etoposide and ifosfamide was examined by flow cytometry.

**Results:**

In vitro as well as ex vivo, the whole mistletoe extract viscumTT led to strong inhibition of proliferation and synergistic apoptosis induction in osteosarcoma cells. In the investigations of mechanism of action, inhibitors of apoptosis such as XIAP, BIRC5 and CLSPN showed a clear down-regulation after viscumTT treatment. In addition, co-treatment with doxorubicin, etoposide and ifosfamide further enhanced apoptosis induction, also synergistically.

**Conclusion:**

ViscumTT treatment results in synergistic apoptosis induction in osteosarcoma cells in vitro and ex vivo. Additionally, conventional standard chemotherapeutic drugs such as doxorubicin, etoposide and ifosfamide were able to dramatically enhance apoptosis induction. These results promise a high potential of viscumTT as an additional adjuvant therapy approach for osteosarcoma.

**Electronic supplementary material:**

The online version of this article (doi:10.1186/s12906-016-1545-7) contains supplementary material, which is available to authorized users.

## Background

Osteosarcoma (OS) is the most common bone tumor with a worldwide incidence of four to five cases annually per million [1]. Nowadays, conventional treatment of osteosarcomas is still surgery with subsequent combined chemotherapy (multidisciplinary therapy). High-grade osteosarcoma tends to develop pulmonary metastasis, and such patients and recurrent patients have a distinctly worse prognosis. Additionally, standard therapies carry the problem of chemotherapy resistance. In the future new adjuvant therapy approaches will be necessary.

*Viscum album* L. extracts (VAEs) are widespread in complementary cancer medicine in Europe and used in several cancer types [2, 3]. *Viscum album* L. includes a large number of chemically different substances: lectins [4], triterpenic acids [5], viscotoxins [6], phenolic acids [7], flavonoids [8], oligo- and polysaccharides [9, 10] and some others. The commercial aqueous VAEs contain mainly mistletoe lectins (MLs) I-III [4] and viscotoxins (VT) [6, 11], and for MLs various biologically activities are known. Cytotoxic and apoptotic potential of MLs have been shown in human lymphocytes [12, 13] and in head and neck squamous cell carcinoma cell lines [14]. VAE treatment led to a significant prolongation of the median overall survival in patients with advanced pancreatic cancer [3]. The basic mechanism of action of *Viscum album* L. is not fully understood. Both the European mistletoe as well as the Korean mistletoe mediated apoptosis induction by activation of the PI3K/AKT pathway [15], JNK/p38/MAPK [16] signalling and caspase cascades [13, 17]. Our group demonstrated apoptosis induction in vitro and in vivo in acute lymphoblastic leukaemia (ALL) [18], in acute myeloid leukaemia (AML) [19] as well as recently in Ewing’s sarcoma (Twardziok et al. accepted). Additionally, down-regulation of inhibitor of apoptosis proteins (IAPs), such as BIRC5 and XIAP was observed in Ewing’s sarcoma cell lines (Twardziok et al. accepted). Activation of the mitochondrial apoptotic pathway was demonstrated inter alia in AML [19], ALL [18] and other cancer cell lines [20].

In addition to MLs and viscotoxins, *Viscum album* L. contains hydrophobic compounds, pentacyclic triterpenic acids, such as oleanolic (OA) and betulinic (BA) acid. Because of their low water solubility they are not included in VAEs. OA and BA possess anti-cancer effects in different tumor entities. OA arrested the cell cycle and led to apoptosis induction in pancreatic [21] and osteosarcoma cells [22] while BA induced mitochondria mediated apoptosis in neuroectodermal tumors [23].

To potentiate all anti-cancer effects of each mistletoe compound, a whole mistletoe extract viscumTT was created by combining mistletoe derived triterpenic acids (TT), which were solubilized by β-cyclodextrins (CD) [24], and a water-based extract (viscum). In recent investigations we and others were able to determine a synergistic apoptosis induction for viscumTT in comparison to the single extracts viscum or TT in leukemia (ALL, AML) [18, 19] and Ewing’s sarcoma (Twardziok et al. accepted) and in murine melanoma in vitro and in vivo [25].

It is already known that adjuvant therapy with VAEs reduces side effects during chemotherapy [26]. Additionally, the impact of various conventional chemotherapeutic drugs can be increased by VAEs as was recently shown in leukaemia cells [27], breast carcinoma and pancreatic cancer cells [28]. The influence of viscumTT co-treatment of osteosarcoma with standard chemotherapy such as doxorubicin (Doxo), etoposide (VP16) and ifosfamide (4OOH) was investigated in this study.

## Methods

### *Viscum album* L. extracts

*Viscum album* L. extracts were kindly provided by Birken AG (Niefern-Oeschelbronn, Germany). Preparation of *Viscum album* L. extracts from apple tree (malus) was performed as described previously [18, 29]. Intact mistletoe lectin I (A + B chain) in viscum extract was analyzed by ELISA [19]. Both the aqueous mistletoe extract viscum and the triterpene extract TT were solubilized in phosphate buffered saline PBS resulting in a final concentration of 2 μg/mL intact ML-I, < 1 μg/mL viscotoxins (viscum), 4000 μg/mL OA and 0.35 μg/mL BA (TT) (Table. 1). As whole mistletoe extract (viscumTT) a combination of the two single extracts (TT and viscum) was added to the cells.

**Table 1:** Composition of viscum, TT and viscum TT extracts

	CDs [mg/mL]	OA [μg/mL]	BA [μg/mL]	ML [ng/mL]	VT [μg/ml]
TT	230	3600	270	/	/
viscum	230	/	/	570	22.13
viscumTT	230	3600	270	570	22.13
CDs	230	/	/	/	/

Represented is the composition of each mistletoe extract, used in the experiments. TT contain mostly oleanolic acid (OA) and betulinic acid (BA) without mistletoe lectin (ML) or viscotoxins (VT). Viscum contains β-cyclodextrins (CDs), ML and VT and does not include OA or BA. ViscumTT is a combination of both single extracts. The concentrations of ML-I as well as OA and BA in each stock solution are shown. The concentrations of ML (for viscum) and OA (for TT) function as marker substances

### Material and reagents

RPMI 1640, McCoy’s 5A, penicillin, streptomycin, trypsin (0.05%) and PBS were purchased from Gibco, Lifetechnologies (Darmstadt, Germany). Fetal Calf Serum (FCS) was obtained from Biochrom (Berlin, Germany). Protein inhibitors, molecular mass standards for SDS-PAGE, sodium dodecyl sulphate (SDS), dimethyl sulfoxide (DMSO), VP16, 5, 5, 6, 6-tetrachloro-1, 1, 3, 3-tetraethylbenzimidazol-carbocyanine iodide (JC-1) and propidium iodide (PI) were purchased from Sigma Aldrich (Munich, Germany). Tween, acrylamide and dithiotreitol (DTT) were purchased from Carl Roth GmbH (Karlsruhe, Germany) and 4-hydroperoxyifosfamide (active metabolite of ifosfamide, 4OOH) was obtained from Niomech (Bielefeld, Germany). Doxo was kindly provided by the hospital pharmacy of the Charité.

### Cell culture

The human osteosarcoma cell lines 143B and Saos-2 were obtained from American Type Culture Collection (ATCC, Manassas, USA). 143B cells were cultured in RPMI 1640, Saos-2 in McCoy’s 5A, both supplemented with 10% heat inactivated FCS 100 U/mL penicillin and 100 μg/mL streptomycin. For the experiments, 143B cells were seeded in 4*10^5^ onto 6 well and 2*10^5^ onto 12 well plates. For Saos-2 cells 1*10^6^ onto 6 well and 5*10^5^ onto 12-well plates were seeded. Cells cultured 24 h to allow cell attachment and treated with viscum (2.5, 5, 10 ng/mL), TT (40, 50, 60 μg/mL) and viscumTT (2.5 + 40, 5 + 50, 10 + 60 ng/mL + μg/mL) 24 and 48 h.

### Proliferation assay WST-1

Cell proliferation reagent WST-1 (Roche, Grenzach-Whylen, Germany) was used for quantification of cell proliferation. 2*10^4^ 143B cells and 5*10^4^ Saos-2 cells were seeded onto 96-well plates in triplicates and incubated 24 and 48 h with viscum, TT and viscumTT in rising concentrations (see Cell culture). 10 μl WST-1 reagent were added and the absorbance of the dye solution was measured after 2 h of incubation in a humidified atmosphere by an ELISA reader (Thermo Fisher Scientific, Bonn, Germany).

### Proliferation measurement and exclusion of early cytotoxicity

Cell number was calculated using a CASY® Cell Counter from Schaerfe System GmbH (Reutlingen, Germany). Alterations of proliferation were expressed in percent in relation to control cells (100% vitality). Lactate dehydrogenase (LDH) release was measured photometrically at 490 nm after 2 h using the Cytotoxicity Detection Kit (Roche, Grenzach-Wyhlen, Germany) to preclude an early cytotoxic effect.

### Detection of apoptosis

Both osteosarcoma cell lines were seeded onto 6 well plates and incubated with increasing concentrations of viscum, TT and viscumTT in rising concentrations (see Cell culture) for 24 and 48 h. For the co-treatment investigations, cells were seeded onto 12 well plates, treated with lower concentrations of viscum (1.5, 2, 2.5 ng/mL), TT (20, 30, 40 μg/mL) and viscumTT (combination in rising concentrations). Additional, VP16 (0.5 μg/mL), Doxo (0.1 μg/mL for 143B cells, 0.05 μg/ml for Saos-2 cells) and 4OOH (0.05 μg/mL) were added and incubated for 48 h. Then cells were washed twice with PBS, re-suspended in 100 μl binding buffer (10 mM HEPES/NaOH, pH 7.4, 140 mM NaCl, 5 mM CaCl_2_) and stained with APC-conjugated Annexin V (BD Bioscience, Heidelberg, Germany) and 1 mg/mL PI according to manufacturer’s instructions to measure apoptosis. The cells were analyzed by flow cytometry (FACSCalibur, Becton Dickinson, Heidelberg, Germany). The results were evaluated with FlowJo Software (TreeStar, Ashland, USA).

### Measurement of mitochondrial membrane potential (∆Ψ_m_)

The ∆Ψ_m_ was measured using JC-1. After incubation, the cells were washed, re-suspended in 750 μl PBS and incubated with 2.5 μg/mL JC-1 for 30 min at 37 °C, 5% CO_2_. After a further PBS washing step the cells were analyzed by flow cytometry. As reference, control cells were treated with 50 μM depolarizing carbonyl cyanide 3-chlorophenylhydrazone (CCCP) (Sigma Aldrich, Munich, Germany) for 30 min.

### Measurement of caspase activity

After an incubation time of 24 h with viscum, TT and viscumTT in rising concentrations (see Cell culture), CASP3, CASP8 and CASP9 activity was assessed using Promokine’s Green Caspase Staining Kit (Promokine, Heidelberg, Germany). For this, cells were incubated with the nontoxic, irreversibly capase-binding cell-permeable FITC-LEHD-FMK, FITC-IETD-FMK and FITC-DEVD-FMK according to manufacturer’s instructions. Then the cells were analyzed by flow cytometry. For caspase inhibitor assay, 143B and Saos-2 cells were pre-incubated with 100 μM pan-caspase inhibitor Z-VAD-FMK for 1 hour and subsequently treated with ≈ IC 50 of TT and viscumTT and ≈ IC 25 of viscum for 24 h. As solvent control DMSO was added. Apoptosis induction was investigated by Annexin V/PI staining and flow cytometry as described earlier.

### Western Blot

For protein extraction 143B cells were incubated with increasing concentrations of viscum, TT and viscumTT for 24 h. The cells were washed twice and lysed using RIPA buffer (Carl Roth GmbH, Karlsruhe, Germany) including proteinase inhibitor for 30 min. Next, the cells were centrifuged at 14,000 rpm for 30 min at 4 °C. Protein concentration was determined by Bradford reagent. After transfer of proteins to nitrocellulose membrane (Bio-Rad, Munich, Germany) primary antibody was incubated over night at 4 °C. The following primary antibodies were used: CASP3 (#9662, Cell Signaling Technology, Danvers, MN, USA), PARP1 (#9542, Cell Signaling Technology), CLSPN (#2800, Cell Signaling Technology), BIRC5 (#2803, Cell Signaling Technology), TP53 (sc-73566, Santa Cruz Biotechnology, CA, USA), XIAP (#610716, BD Biosciences), BCL2 (#2870, Cell Signaling Technology), BID (#2002, Cell Signaling Technology), cytochrome c (#4280, Cell Signaling Technology), ß-actin (#A3854, Sigma-Aldrich) and GAPDH (sc25778, Santa Cruz Biotechnology, CA, USA). Protein signal was detected by HRP-conjugated secondary antibodies (Bio-Rad, Munich, Germany) and visualized by ECL (Thermo Fisher Scientific, Bonn, Germany) and ChemiDoc. For mitochondrial protein preparation, cells were lysed as described above and the cytosolic fraction was isolated as published earlier [18]. Then, the cytosolic fraction was seperated by SDS-PAGE, transferred to nitrocellulose membrane and incubated with monoclonal cytochrome c antibody. Actin was used as loading control.

#### ex vivo cultured osteosarcoma primary cells

After routine surgical resection, a tumor sample from the tibia was obtained as treatment ‘residue’ from a 12-year-old boy with first-diagnosis osteosarcoma without lung metastases. This tumor material was not explicitly collected for this research. Histopathology confirmed the diagnosis. Immediately after surgical excision, the tissue was dissected into smaller pieces and cultured as a primary explant in RPMI 1640 base medium with L-glutamine supplemented with 20% heat-inactivated FCS and 1% penicillin/streptomycin solution. When cells dissociated from the explant to form a confluent monolayer culture ex vivo cells were treated in 4 trypsinized passages. For investigations, cells were seeded onto 12-well microtiter plates at 2.5×10^5^/well for treatment with viscum, TT or viscumTT extracts. For each tested dose, single well plate was used and experiment was performed at least in three sets of independent experiments. Then, proliferation, apoptosis induction, activation of CASP8, CASP9, CASP3 and loss of ∆Ψ_m_ were examined. Written informed consent was obtained from the patient in accordance with the Declaration of Helsinki, and the study was approved by the local ethics committee of the Charité- Universitätsmedizin Berlin.

#### Statistics

All in vitro experiments were performed at least in three sets of independent experiments, for which means ± standard error were calculated and plotted in bar graphs. Webb’s fractional product (*Fp) >1 was calculated for the synergistic effect of viscumTT and the chemotherapeutic agents on apoptotic induction in vitro as described earlier [18, 30]. Briefly, Webb’s fractional product based on the formula: E_1;2exp_ = (E_1_ + E_2_-E_1_*E_2_), with E_1;2exp_ = expected (calculated) effect of the combination viscumTT. E_1_ = observed effect of TT; E_2_ = observed effect of viscum; E_1;2obs_ = observed effect of combined mixture viscumTT. Fp = E_1;2obs_/E_1;2exp._ Values Fp > 1 represent synergistic, whereas Fp = 1 additive and Fp < 1 antagonistic effects. One-way ANOVA was used for calculation of significance. All results with p ≤ 0.05 were considered significant.

## Results

### Viscum, TT and viscumTT did not induce early cytotoxicity but inhibit cell proliferation in vitro

**Fig. 1:**
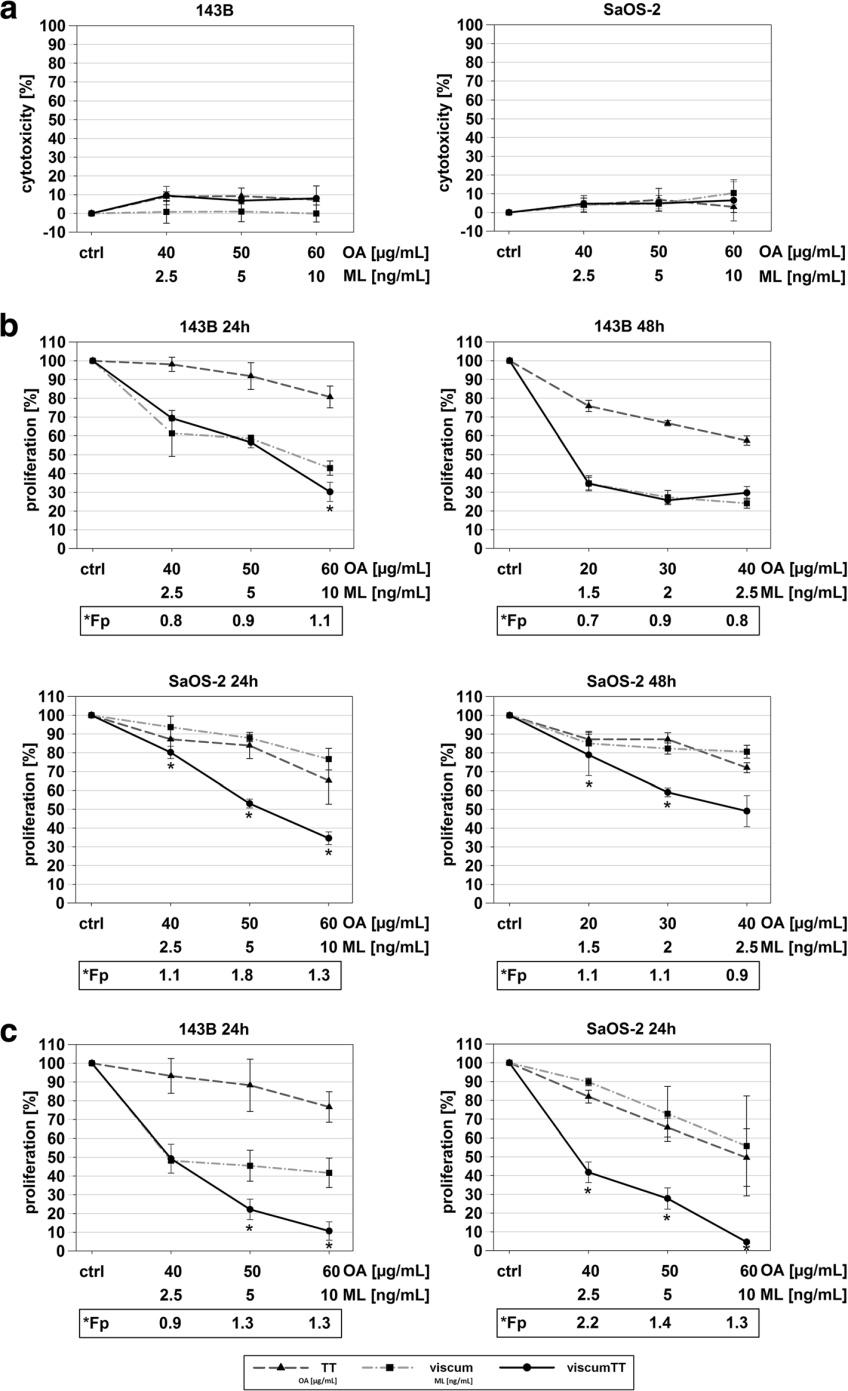
Viscum, TT and viscumTT inhibit proliferation without early cytotoxicity in 143B and Saos-2 cells*.* Early cytotoxicity was analyzed via LDH release into the culture medium (**a**). Proliferation was examined by CASY® Cell Counter (**b**) and WST-1 assay (**c**). All results are presented as percentage of untreated control (Ctrl). Control cells were set to 100%. Means ± SD are shown. Mistletoe lectin (ML) and oleanolic acid (OA) concentrations were used as a measure of viscum and TT active agent extract concentration. (*p* ≤ 0.05, *n* ≥ 3, *Fp > 1 = synergism)

Early cytotoxicity was analyzed by Cytotoxicity Detection Kit after 2 h whereby LDH release was investigated. Neither 143B nor Saos-2 cells showed any considerable increase of LDH in the supernatant (Fig. 1a). Therefore, an unwanted early cytotoxicity via necrosis could be excluded.

For assessment of inhibition of proliferation, cells were counted using a CASY® Cell Counter and proliferation was estimated from total numbers of cells compared to control cells. Inhibition of proliferation was confirmed by WST-1 assay. Both cell lines showed strong inhibition of cell proliferation in a dose-dependent manner after treatment with viscum, TT and viscumTT after 24 h and 48 h (Fig. 1b and c). TT inhibited cell proliferation less than viscum. For viscumTT the effect was enhanced in both cell lines. With the WST-1 assay a synergistic effect could be determined.

### ViscumTT induces apoptosis in both cell lines synergistically

**Fig. 2:**
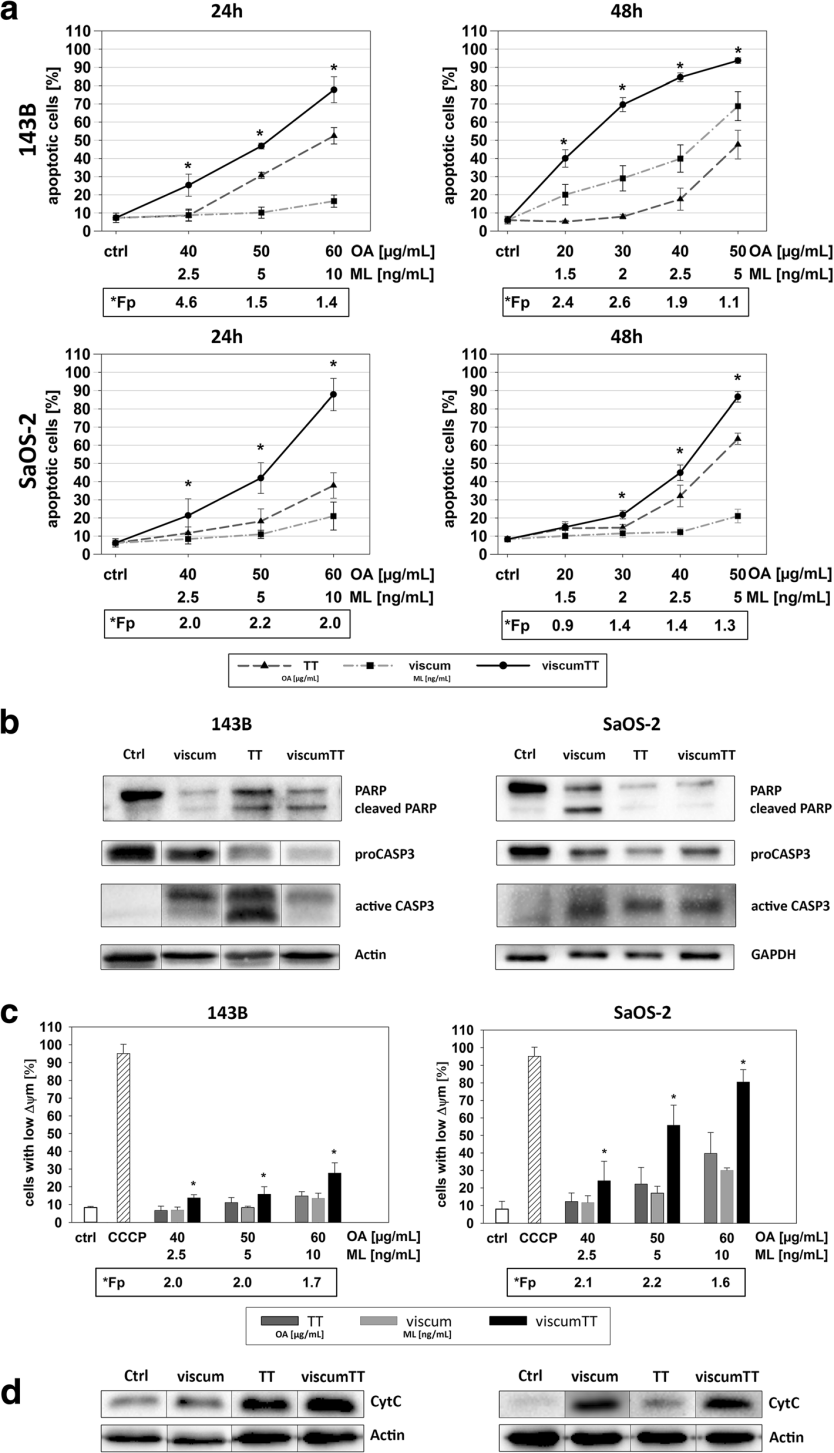
ViscumTT induces apoptosis synergistically and leads to loss of mitochondrial membrane potential (∆Ψ_m_). The means ± SD of apoptotic cells are shown (**a**). Mistletoe lectin (ML) and oleanolic acid (OA) concentrations were used as a measure of viscum and TT. (*p* ≤ 0.05, *n* ≥ 3, *Fp > 1 = synergism). Apoptosis was confirmed by proteolytic processing of CASP3 and cleavage of PARP (**b**). β-actin was used as loading control. The means ± SD of loss of mitochondrial membrane potential (∆Ψ_m_) (**c**) and carbonyl cyanide 3-chlorophenylhydrazone (CCCP was used as positive control (*p* ≤ 0.05, *n* ≥ 3, *Fp > 1 = synergism) are shown. Cytochrome c (**d**) release confirmed mitochondrial pathway activation. β-actin served as loading control

Next, the potential of viscum, TT and viscumTT for apoptosis induction was determined. 143B and Saos-2 cells were analyzed by annexin V-APC/PI staining and flow cytometry after 24 and 48 h treatment. Interestingly, viscum led to only a slight apoptosis induction in both cell lines, whereas TT and viscumTT triggered apoptosis in a dose- and time-dependent manner after 24 h (Fig. 2a). This effect was enhanced after 48 h (Fig. 2a) for all three extracts. ViscumTT led even to a synergistic apoptosis induction in both osteosarcoma cell lines (Fig. 2a). The apoptosis induction was confirmed by cleavage of PARP and proteolytic processing of CASP3 after western blot analysis (Fig. 2b).

### Viscum, TT and viscumTT induce mitochondrial depolarization and cytochrome c release

For investigation whether mitochondria are involved in the apoptotic process, the Δψm depolarization was evaluated by JC-1 and FACS analyses after 24 h extract treatment. ViscumTT treatment led to a significant dose-dependent loss of Δψm (Fig. 2c) and confirmed the synergistic effect observed in annexin V staining. In 143B cells the observed effect was much less for all three extracts in comparison to Saos-2 cells.

Δψm depolarization and resulting cytochrome c release into the cytosol mediate apoptosis induction via the intrinsic pathway. Based on western blot analyses cytochrome c was detected in the cytosol fraction after treatment with viscum and viscumTT (Fig. 2c) in 143B and additionally after TT in Saos-2. These results suggest the hypothesis that viscum, TT and viscumTT induce apoptosis via the mitochondrial pathway.

### Viscum TT leads to activation of CASP8 and CASP9

**Fig. 3:**
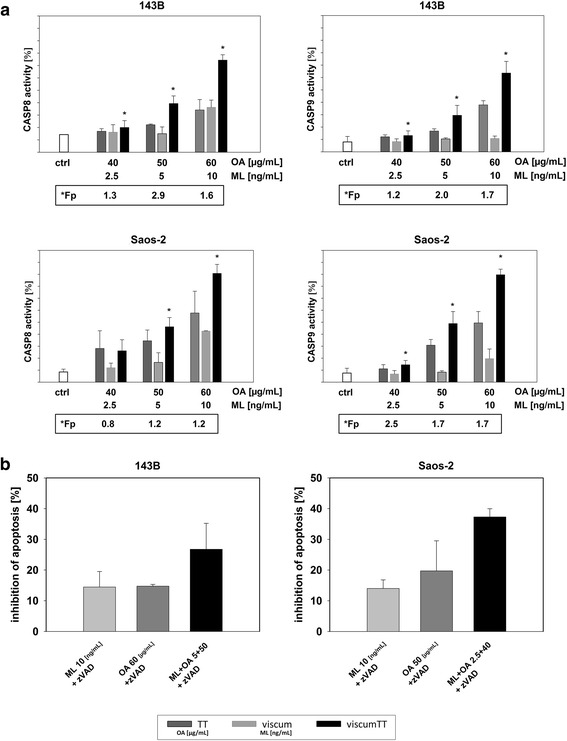
ViscumTT enhances activation of CASP8 and CASP9. FITC-LEHD-FMK and FITC-IETD-FMK staining and FACS analyses represented activity of CASP8 and CASP9 (**a**). Pre-incubation with ZVAD-FMK pan-caspase inhibitor confirmed caspase-dependent apoptosis induction (**b**) (mean ± SD, *p* ≤ 0.05, *n* ≥ 3, *Fp > 1 = synergism)

To get a closer look at the apoptosis mechanism, we assessed the activation of CASP8 and CASP9. These caspases are upstream initiator protease caspases, which will be activated by the extrinsic (CASP8) and intrinsic (CASP9) apoptotic pathways and initiates cleavage of effector caspase CASP3. CASP3 and the presence of cytochrome c can additionally induce cleavage of CASP8 as a feedback mechanism without receptor mediation [31]. CASP8 was affected by viscum and TT alone in both cell lines (Fig. 3a). CASP9 (Fig. 3a) was activated after the treatment with TT and viscumTT but viscum alone had practically no effect, while viscumTT treatment led to clearly synergistic activation of both caspases. After pre-incubation for 1 h with ZVAD-FMK pan-caspase inhibitor apoptosis induction was significantly reduced up to 40% in both cell lines treated with viscumTT and up to 10 to 15% in both cell lines treated with viscum and TT (Fig. 3b). These results validated the essential role of caspases in apoptosis induction by viscumTT.

### ViscumTT alters apoptosis associated proteins

**Fig. 4:**
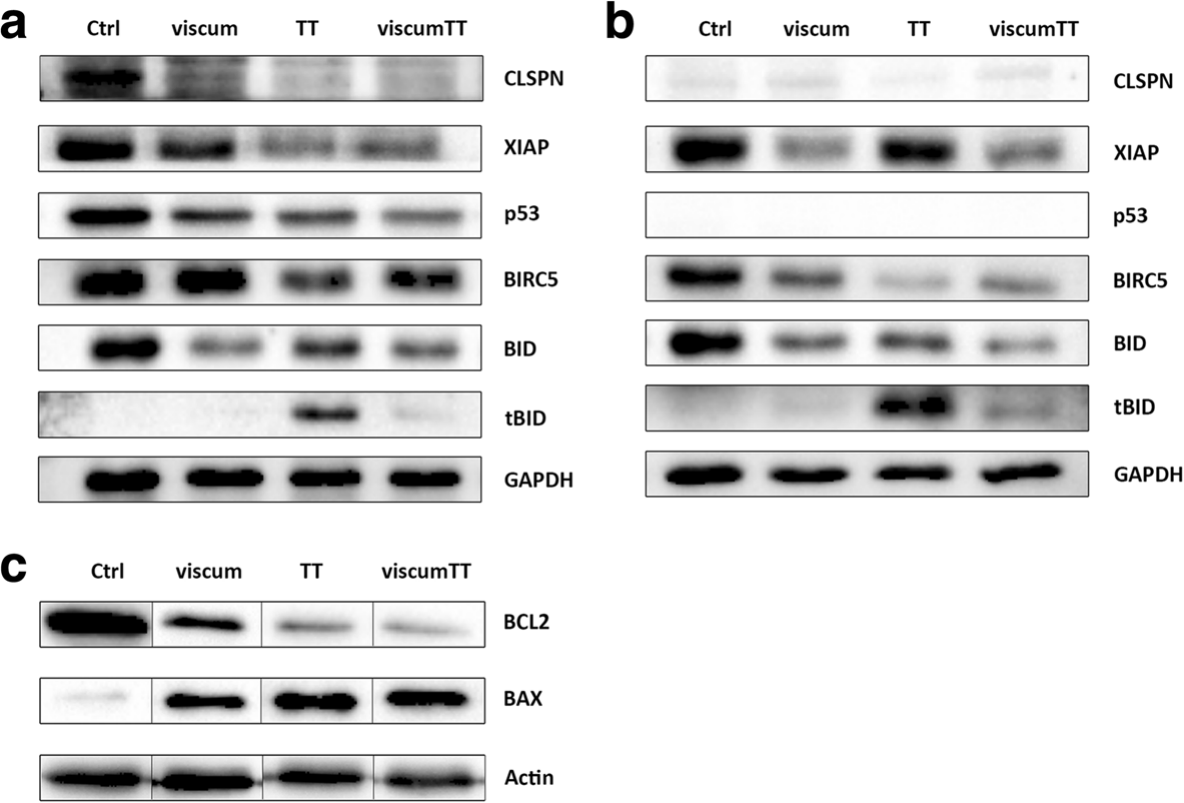
Viscum, TT and viscumTT alter apoptosis associated protein expression levels. Western blots of p53, BCL-2, BAX and BID as well as XIAP, CLSPN and BIRC5 after viscum, TT and viscumTT treatment in 143B (**a, c**) and SaOs-2 (**b**) cells. GAPDH and β-actin served as loading controls (*n* ≥ 3)

Because of previous knowledge, we wanted to know which proteins of the bcl-2 family and inhibitor of apoptosis proteins (IAPs) are altered in osteosarcoma. All three extracts led to down-regulation of anti-apoptotic protein BCL2 with simultaneous up-regulation of pro-apoptotic protein BAX in 143B cells (Fig. 4c). A reduced expression of XIAP and BIRC5 was observed in both cell lines (Fig. 4a and b). Interestingly, the expression of CLSPN was strongly down-regulated by viscum, TT and viscumTT in 143B cells, whereas in SaOS-2 control cells CLSPN is not expressed at all. Additionally, decreased BID expression with cleavage of BID into tBID after TT treatment was also observed (Fig. 4a and b). p53 was down-regulated by viscum, TT and viscumTT in 143B.

### Synergistic effect of viscumTT is confirmed in patient-derived osteosarcoma cells

**Fig. 5:**
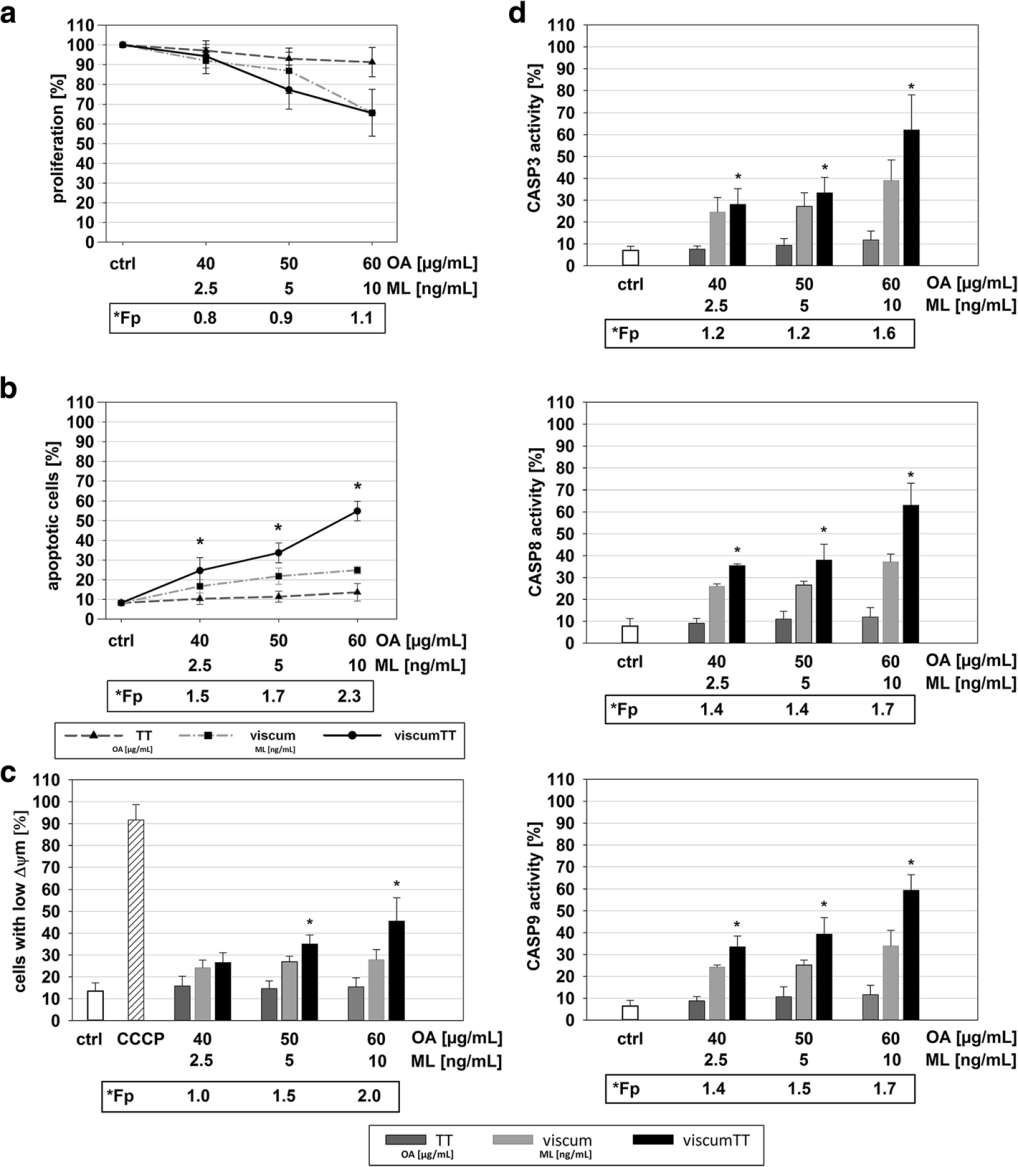
ViscumTT synergistically induces apoptosis in primary material from an osteosarcoma patient**.** Inhibition of proliferation (**a**), apoptosis induction (**b**), loss of mitochondrial membrane potential (∆Ψ_m_) (**c**) and CASP8, CASP9 and CASP3 (**c**) activity are shown as mean % ± SD. Proliferation was measured by CASY® cell counter and control was set to 100%. Alterations of proliferation were expressed in % of control cells (*p* ≤ 0.05, *n* ≥ 3, *Fp > 1 = synergism)

To investigate whether there is any apoptotic effect on primary tumor material, derived osteosarcoma cells were treated with viscum, TT and viscumTT. Figure 5 shows inhibition of cell proliferation (A) with further induction of apoptosis (B) and loss of Δψm (C). Additionally, activation of CASP3, −8 and −9 (D) was synergistically activated after viscumTT treatment. These results confirmed our in vitro data.

### Co-treatment with chemotherapeutic drugs increases apoptotic response

**Fig. 6:**
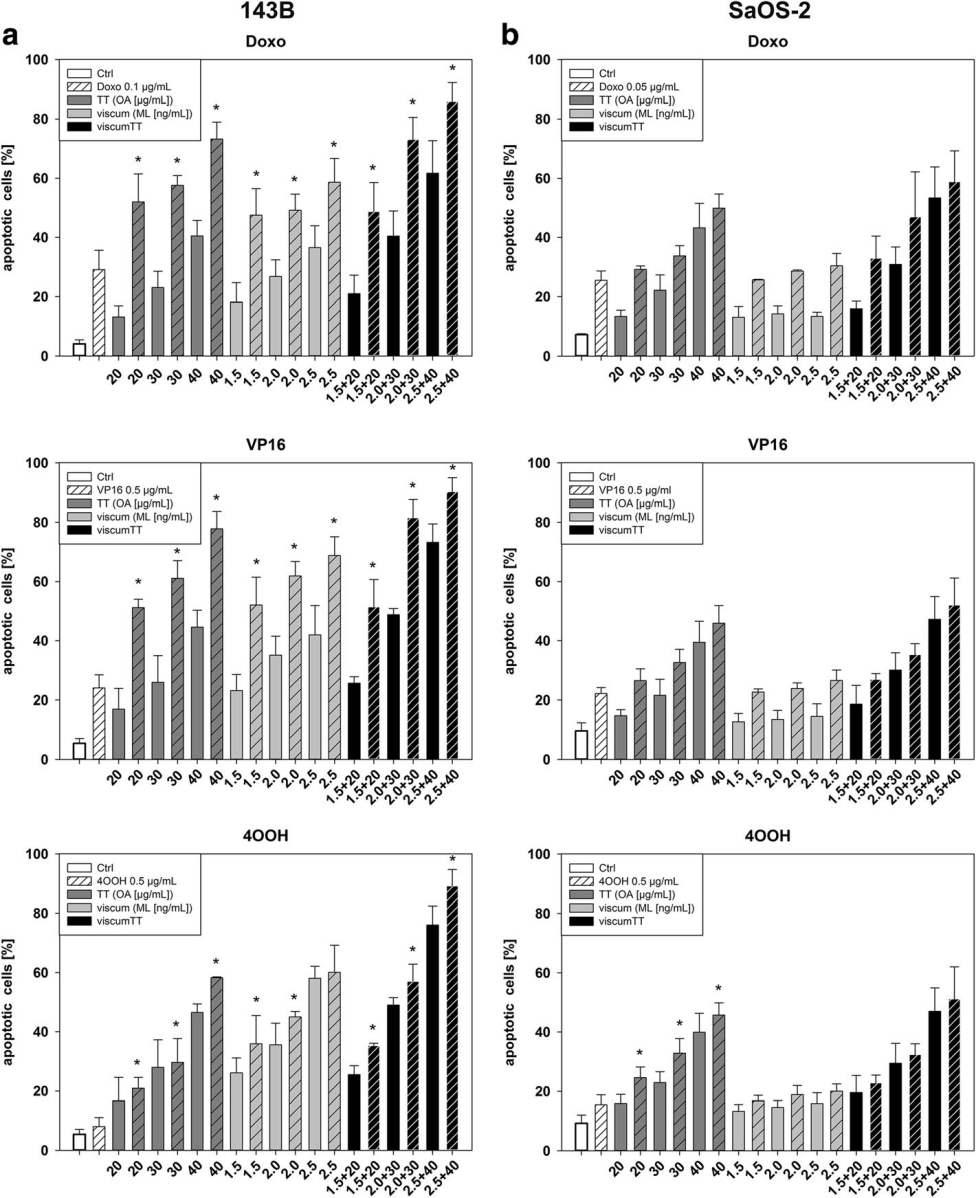
ViscumTT co-treatment with doxorubicin (Doxo), etoposide (VP16) and ifosfamide (4OOH) enhances apoptosis induction synergistically. FACS analyses of apoptotic cells in % ± SD of 143B (**a**) and SaOS-2 (**b**) cells. Mistletoe lectins (MLs) and oleanolic acid (OA) were used as marker substances for viscum and TT (*p* ≤ 0.05, *n* ≥ 3, *Fp > 1 = synergism)

Because of the increasing presence of resistances to conventional chemotherapy in osteosarcoma a combination treatment with complementary therapy approaches is becoming more and more important. Beside treatment with viscum, TT and viscumTT, osteosarcoma cell lines were co-treated with Doxo, VP-16 and 4OOH to examine apoptosis induction. In both cell lines apoptosis was induced even when TT and viscum alone were added to chemotherapeutic drugs. This effect was additionally enhanced in 143B after co-treatment with viscumTT (Fig. 6a). In Saos-2 cells (Fig. 6b) the enhancer effect of viscumTT is rather low. The strongest synergistic effect was observed when VP16 or Doxo was combined with viscumTT in a lower dose. Higher dosage led to an additive effect (Table 2).

**Table 2:** Webb’s fractional product of co-treatment of chemotherapy with viscum, TT and viscumTT

	TT			viscum			viscumTT		
A									
DOXO	0.1 + 20	0.1 + 30	0.1 + 40	0.1 + 1.5	0.1 + 2	0.1 + 2.5	0.1 + 1.5 + 20	0.1 + 2 + 30	0.1 + 2.5 + 40
*FP	1.39	1.37	1.32	1.22	1.07	1.11	1.06	1.32	1.26
VP16	0.5 + 20	0.5 + 30	0.5 + 40	0.5 + 1.5	0.5 + 2	0.5 + 2.5	0.5 + 1.5 + 20	0.5 + 2 + 30	0.5 + 2.5 + 40
*FP	3.20	2.48	1.73	2.44	1.86	1.72	2.00	1.79	1.27
4OOH	0.5 + 20	0.5 + 30	0.5 + 40	0.5 + 1.5	0.5 + 2	0.5 + 2.5	0.5 + 1.5 + 20	0.5 + 2 + 30	0.5 + 2.5 + 40
*FP	1.14	1.10	1.28	1.37	1.20	0.97	1.19	1.13	1.20
B									
DOXO	0.05 + 20	0.05 + 30	0.05 + 40	0.05 + 1.5	0.05 + 2	0.05 + 2.5	0.05 + 1.5 + 20	0.05 + 2 + 30	0.05 + 2.5 + 40
*FP	0.83	0.82	0.82	0.72	0.84	0.85	0.80	0.89	0.83
VP16	0.5 + 20	0.5 + 30	0.5 + 40	0.5 + 1.5	0.5 + 2	0.5 + 2.5	0.5 + 1.5 + 20	0.5 + 2 + 30	0.5 + 2.5 + 40
*FP	0.99	1.00	0.94	0.85	0.89	1.01	0.85	0.84	0.93
4OOH	0.5 + 20	0.5 + 30	0.5 + 40	0.5 + 1.5	0.5 + 2	0.5 + 2.5	0.5 + 1.5 + 20	0.5 + 2 + 30	0.5 + 2.5 + 40
*FP	1.22	1.23	1.04	0.75	0.85	0,86	0.85	0.92	0.91

Co-treatment of viscum (ng/mL), TT (μg/mL) and viscumTT with doxorubicin (Doxo (0.1 (A), 0.05 (B) μg/mL)), etoposide (VP16 (0.5 μg/mL)) and ifosfamide (4OOH (0.5 μg/mL)) led to synergistic or additive apoptosis induction in 143B (A) and Saos-2 (B) cells (*Fp > 1 = synergistic effect, *Fp = 1 = additive effect). Oleanolic acid (OA) and mistletoe lectin (ML) were used as marker substances for viscum and TT

## Discussion

In this study we investigated the apoptotic potential of the whole mistletoe extract viscumTT in comparison to its constituents viscum and TT. ViscumTT very effectively inhibited cell proliferation and synergistically induced apoptosis in osteosarcoma cells in vitro in a time- and dose-dependent manner. Our in vitro data correlate with the assumption that whole plant extracts are often more potent in anti-cancer activity than the single ingredients [32, 33]. The mechanisms of action of the individual constituents are still unclear. We and others were able to demonstrate a caspase-dependent apoptosis induction with involvement of CASP8, CASP9 and CASP3 [19, 34]. Bantel *et al.* proved the activation of CASP8 without any death receptor signalling [17], which supported the assumption that apoptosis is mainly mediated by the mitochondrial pathway. CASP3 [35] and cytochrome c [31] can also activate CASP8 in a feedback mechanism. Further cleavage of BID by CASP8 triggers a stronger apoptotic response by an intrinsic mechanism. We observed activation of CASP3, CASP8 and CASP9 with additional loss of mitochondrial membrane potential, BID cleavage and cytochrome c release after viscumTT treatment. TT treatment led to no cytochrome c release in Saos-2 although CASP9 was activated (Figs. 2 and 3). Both cell lines are almost resistant to viscum after 24 h and in 143B cells CASP9 was interestingly not affected. Older studies of our group observed CASP9 and CASP8 activation after viscum treatment in different leukaemia cell lines [18, 19]. However, after addition of TT CASP9 was strongly activated in both cell lines. These results support the statement that the anti-cancer effect is often dependent on cell line and tumor entity. In addition, different OA derivatives induce apoptosis directly mediated by CASP8 in various cancer cells [23, 36, 37]. Apoptosis induction by VAE alters apoptosis associated protein levels of the BCL2 family proteins BAX and BCL2 [38, 39]. This is in line with our results. p53 interacts directly with BCL-2 family members [40] and regulates the expression of BAX and BCL2 in apoptosis [41, 42]. p53 was down-regulated by all three extracts in 143B. In hepatocarcinoma cells [38] and human lymphocytes [39] down-regulation of p53 was also observed. Saos-2 cells are null mutants for p53 but apoptosis was also induced after treatment. These results suggest the assumption that apoptosis induction is p53 independent in these osteosarcoma. Cell lines. Furthermore, VAEs led to reduction in several IAP family members such as XIAP and BIRC5 in Ewing sarcoma (Twardziok et al. accepted)), AML [19], colon cancer and epidermoidal cancer [43]. This is in line with our present results. The abilities of IAPs to inhibit caspases, assemble pro-apoptotic protein complexes and mediate expression of anti-apoptotic proteins, make them promising targets in cancer treatment [44]. Interestingly, CASP3 is able to degrade its own inhibitor (XIAP) which again enhances CASP3 activity and consequently induces apoptosis [45]. In addition, XIAP and BIRC5 are also affected by derivatives of OA [46, 47] and BA [48]. It is evident from our results that viscumTT combines the anti-cancer effects cell type independently, but seems to modulate the mechanism of apoptosis derived from triterpene acids and mistletoe lectins. The mechanism of the synergistic action of viscumTT could not yet be decoded. Recently, it was shown that after a short incubation time uptake of ML by 143B cells after viscum treatment only occurred after addition of TT [49]. Our data suggest that ML uptake needs more time in 143B cells because after 24 h an anti-proliferative effect was observed. However, this observation leads to the hypothesis that triterpenic acids improve the uptake of viscum, which could also be one explanation for the synergistic effect. Triterpenic acids are able to embed in cell membranes, which leads to their disturbance [50, 51], and interact with anionic phospholipids [52]. Further investigations of this are necessary. In the future, reduction of side effects and overcoming of resistances during chemotherapy will be of increasing interest. Some studies were even able to show an enhancement of cytotoxicity when chemotherapy was combined with VAE in vitro [28]. An optimal anti-tumor effect was demonstrated in K562 leukaemia cells after co-treatment with VAE and sub-apoptotic doxorubicin concentrations [27]. Also, VP16 led to an enhanced apoptosis induction after combined incubation with ML-I [17]. In our study we observed an additional synergism when viscum, TT and viscumTT were co-treated with Doxo, 4OOH and VP16 in 143B cells. In Saos-2 cells the enhanced apoptotic effect was additive rather than synergistic. The synergistic apoptosis induction after co-treatment with viscum, TT and viscumTT and 4OOH was demonstrated here for the first time. Generally, hight potential of apoptosis induction, also in combination with classical chemotherapeutic drugs, promises importance as additional therapy in osteosarcoma.

## Conclusion

In this study we demonstrated the high potential of viscumTT with regard to apoptosis induction in osteosarcoma. It induced apoptosis synergistically. This effect is further enhanced when it is co-administered with Doxo, VP16 and 4OOH. Because of increasing problems during chemotherapy such as resistances or negative side effects, new therapeutic approaches will become more important in the future. Hence, viscumTT may represent a promising adjuvant therapy in pediatric osteosarcoma.

## Additional file

Additional file 1: Mean and standard deviation of the single values for each graph. (XLS 106 kb)

## Abbreviations

4OOH: ifosfamide
BA: Betulinic acid
CD: β-cyclodextrin
Doxo: Doxorubicin
LDH: Lactate dehydrogenase
ML: Mistletoe lectin
OA: Oleanolic acid
PBS: Phosphate-buffered saline
TBST: Tris-buffered saline with Tween-20
TT: Triterpene acid-containing extract
VAE: *Viscum album* L. extract
VP16: etoposide
